# Could estrogen impact a new pertinent gene for AIS?

**DOI:** 10.1186/1748-7161-10-S1-O2

**Published:** 2015-01-19

**Authors:** Florina Moldovan, Amani Hassan, Edward Bagu, Charlotte Zaouter, Shunmoogum A Patten

**Affiliations:** 1 Research Center, Sainte-Justine University Hospital Center, Montréal, Canada; 2 Department of Stomatology, Faculty of Dental Medicine, Université de Montréal, Canada; 3 Department of Biochemistry, College of Medicine, University of Saskatchewan, Saskatoon, Saskatchewan, Canada; 4 CRCHUM and Department of Neuroscience, Université de Montréal, Canada

## Introduction

Adolescent Idiopathic Scoliosis (AIS) is a complex rotational spinal deformity that occurs during the pubertal growth spurt. Recently, through a stepwise association study a new susceptibility locus on chromosome 6q24.1 was reported in Japanese population. The most significantly associated SNP, rs6570507, was in GPR126. This gene is coding for a protein of the adhesion subfamily of G-protein coupled receptors. We identified variants in another orphan members of the adhesion subfamily of G-protein coupled receptors which that is characterized by a long serine/threonine-rich N-terminus possibly regulated by hormones such as estrogens and consequently involved in the progression of AIS during the pubertal growth. The aim of this study was to investigate the regulation of this gene (GPRCh3) by 17-beta-estradiol.

## Methods

*In-silico* analysis for potential ERE sites in the GPRCh3 promoter was done using MatInspector and ECR-Browser and then several promoter fragments were cloned in PGL3 vector upstream of the luciferase gene. Huh-7 cells were then transiently transfected with the GPR128 promoter constructs. The luciferase activity was measured in the presence or absence of 17-beta-estradiol. RNA was extracted and qPCR was performed on osteoblasts that is overexpressing the estrogen receptor hfob/ER9 as well as in Huh-7 cells that were either treated with 17-beta-estradiol or Vehicle.

## Results

In Huh7 hepatic cell lines that were transfected with the GPRCh3 promoter constructs, treatment with 17-beta-estradiol over a period of 24 hours following over-expression of ER-α led to a 2.5 fold increase in the promoter activity, confirming the regulation of GPRCh3 by estrogen. Likewise the expression of GPRCh3 mRNA was increased by 3-fold following treatment of Huh7 cells with 17-beta-estradiol.

## Conclusion

Our study demonstrated that estrogen is involved in the expression of GPRCh3. These results could help to understand the molecular mechanisms involved in AIS pathogenesis.

